# A mass and charge balanced metabolic model of Setaria viridis revealed mechanisms of proton balancing in C4 plants

**DOI:** 10.1186/s12859-019-2941-z

**Published:** 2019-06-27

**Authors:** Rahul Shaw, C. Y. Maurice Cheung

**Affiliations:** Yale-NUS College, 16 College Avenue West, Singapore, 138527 Singapore

**Keywords:** Setaria viridis, C4 photosynthesis, Genome-scale metabolic network model, Bioenergy grasses, Lignocellulosic biomass, Gene association, Ammonium and nitrate usage, Mass and charge balance, Millet

## Abstract

**Background:**

C4 photosynthesis is a key domain of plant research with outcomes ranging from crop quality improvement, biofuel production and efficient use of water and nutrients. A metabolic network model of C4 “lab organism” *Setaria viridis* with extensive gene-reaction associations can accelerate target identification for desired metabolic manipulations and thereafter in vivo validation. Moreover, metabolic reconstructions have also been shown to be a significant tool to investigate fundamental metabolic traits.

**Results:**

A mass and charge balance genome-scale metabolic model of *Setaria viridis* was constructed, which was tested to be able to produce all major biomass components in phototrophic and heterotrophic conditions. Our model predicted an important role of the utilization of NH$_{4}^{+}$ and NO$_{3}^{-}$ ratio in balancing charges in plants. A multi-tissue extension of the model representing C4 photosynthesis was able to utilize NADP-ME subtype of C4 carbon fixation for the production of lignocellulosic biomass in stem, providing a tool for identifying gene associations for cellulose, hemi-cellulose and lignin biosynthesis that could be potential target for improved lignocellulosic biomass production. Besides metabolic engineering, our modeling results uncovered a previously unrecognized role of the 3-PGA/triosephosphate shuttle in proton balancing.

**Conclusions:**

A mass and charge balance model of *Setaria viridis*, a model C4 plant, provides the possibility of system-level investigation to identify metabolic characteristics based on stoichiometric constraints. This study demonstrated the use of metabolic modeling in identifying genes associated with the synthesis of particular biomass components, and elucidating new role of previously known metabolic processes.

**Electronic supplementary material:**

The online version of this article (10.1186/s12859-019-2941-z) contains supplementary material, which is available to authorized users.

## Introduction

Plants belonging to the millet species have been cultivated in many developing countries as the important source of calories. *Pennisetum glaucum* (Pearl millet or ‘Bajra’) and *Eleusine coracana* (finger millet or ‘Ragi’) have been used in Indian subcontinent (also in Africa), mostly in rural regions as the low cost staple food crops [1, 2]. Beside millet, important crops like maize (*Zea mays*), sugarcane (*Saccharum officinarum*) and *Sorghum* of tribe *Andropogoneae* and bioenergy crop switchgrass (*Panicum virgatum*) also use C4 photosynthesis [3], where plants fix CO_2_ into a C4 compound in the mesophyll (M) cells before transporting the C4 compound to the bundle sheath (BS) cells where it is decarboxylated to generate a high CO_2_ concentration which minimizes oxygenase activity of rubisco [3, 4]. C4 photosynthesis allows plants to use nitrogen and water more efficiently than C3 which helps in their high rates of productivity [5, 6]. Thus, research on C4 plants has potential applications in improving worldwide cereal yield [7], modification and utilization of unused plant parts as biofuel feedstock [8, 9] and to provide directions to engineer C4 pathway in C3 species like ‘C4 rice’ to improve grain biomass [10]. Importantly, all these research domains need a model lab organism for C4 plants. *S. viridis* uses the NADP-ME (NADP-malic enzyme) subtype of C4 pathway which is also used by many important C4 crop plants like maize, sorghum, and sugarcane and bioenergy feedstock *Miscanthus x giganteus* [3]. *S. viridis* or green millet is a close weedy relative of domesticated *Setaria italica* (foxtail millet) [4]. *S. italica* has been used as the model to study bioenergy grasses thus, as a domesticated crop, it is an advantageous biofuel feedstock [9, 11]. *S. viridis* has some advantages over *S. italica* regarding its application in biological research like having small diploid genome, short height (30 cm at maturity), shorter harvest time (6-8 weeks) and distinct advantages for forward and reverse genetics and high throughput phenotyping [3]. With these advantages, *S. viridis* has the potential to obtain similar reputation among researchers as a lab organism for the study of C4 photosynthesis as *Arabidopsis thaliana* has for C3 plants.

In this study, we constructed a mass and charge balanced genome-scale metabolic model (GSM) of *S. viridis* as a tool for C4 systems biological research. GSMs were built in an attempt to explore complex multi-cell interactions of C4 photosynthesis. C4GEM, the first set of genome-scale models of C4 plant metabolism, was utilized to explore the metabolic interactions between two different cell types observing classical C4 photosynthesis and its complex interaction in M and BS cells [12]. Recently, a modeling framework, MultiGEM, was developed to model the spatiotemporal metabolic behavior which was applied to identify reactions and pathways that correlates with improved polyhydroxybutyrate (PHB) yield [13]. A GSM of *S. italica* was developed and applied to analyze plant developmental stages and their different levels of regulation [14].

Here, we applied GSM of *S. viridis* to study the balancing of protons (H ^+^) and charges in C4 plants. H ^+^ gradient across the thylakoid membrane has elemental role in ATP synthesis [15]. H ^+^ flow mechanism also serves to prevent photodamage by regulating light capture by the photosynthetic antenna and can also regulate ATP/NADPH output ratio [16]. Overall plant performance greatly depends on H ^+^ gradients maintained by H ^+^-pumps contributing to plant chemiosmotic circuits [17]. A reduction in proton-motive force has also been shown to affect root growth [18]. Besides H ^+^ gradients, charge balancing has been shown to be important in plants, e.g. anion uptake stimulates cation uptake and translocation, which is used to balance organic anions on nitrate (NO$_{3}^{-}$) reduction in leaves [19]. Cation-anion uptake and their balance along with soil condition can determine Fe nutrition and rhizosphere pH by H ^+^ excretion [20]. Cation vs anion uptake also determines soil acidification [21] whereas internal H ^+^ and OH ^−^ fluxes can regulate cytoplasmic pH [22]. Recently a metabolic model representing central plant metabolism of CAM plants used all charge and H ^+^ balanced reactions as well as multiple charged form of a metabolite to account for pH in different compartments [23]. This enables the energetics and productivity analysis of CAM metabolism under acidification of vacuole, i.e. to observe the significance of H ^+^ balancing. This highlights the importance of using complete mass, charge and H ^+^ balanced GSMs for the mining of inherent metabolic features which are coupled with ion pools.

While the main process of C4 photosynthesis are now relatively well understood, there are still some evolutionary, molecular, regulatory features and bioengineering prospect which are yet to be identified (see [24] and references therein). The GSM of *S. viridis*, with extensive gene-reaction association, can contribute to these omni-directional C4 research in terms of hypothesis generation and testing. To demonstrate such applications of a large-scale mass and charge balance metabolic model, we used our model to identify reactions and genes involved in the production of three main components of lignocellulosic biomass. During this process, our modeling results highlighted a potential role of cation-anion balancing between the two cell types in the C4 photosynthetic pathway.

## Materials and method

### Construction of a genome-scale metabolic model of *S. viridis*

**Fig. 1:**
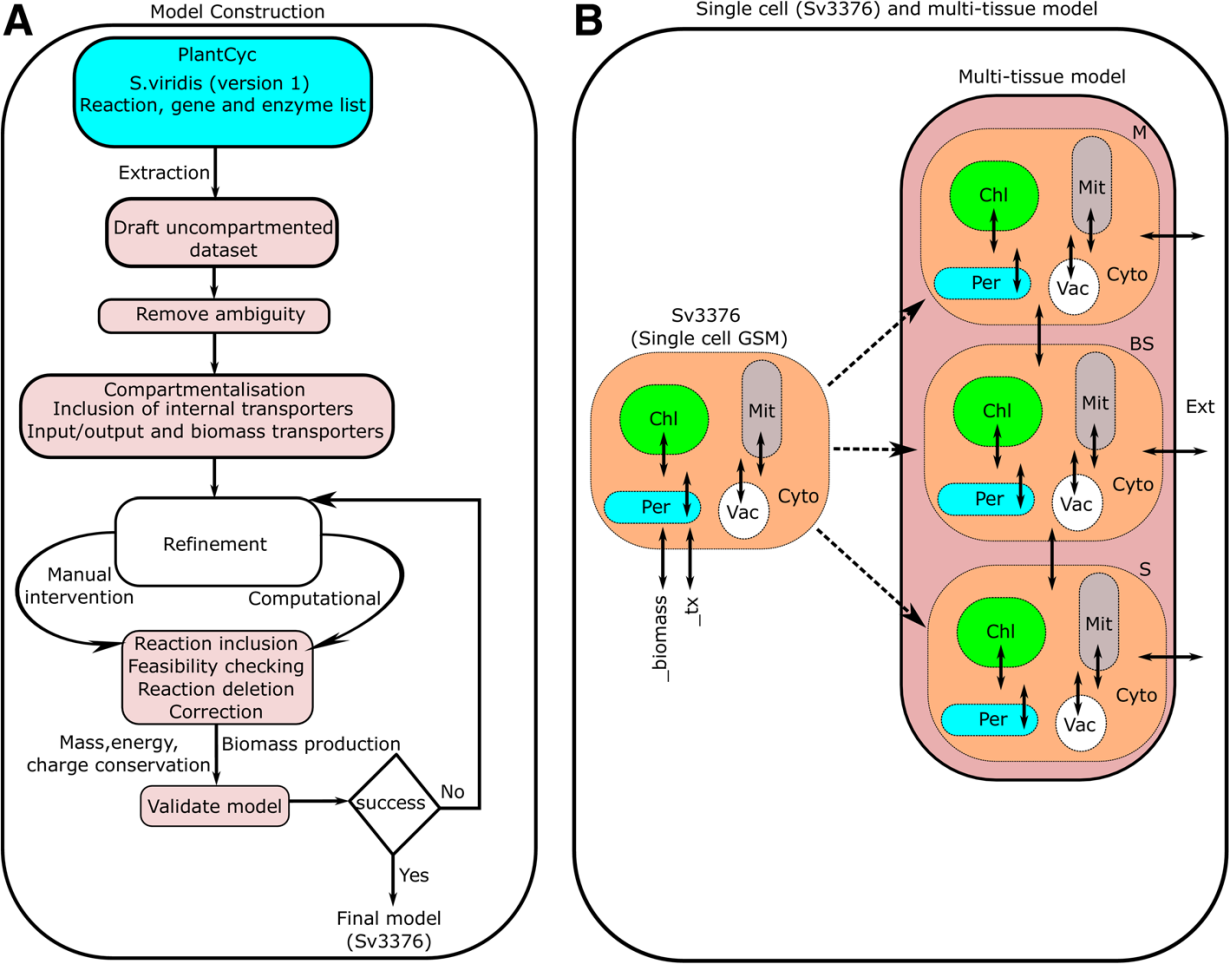
**a** Flowchart of model construction process from PlantCyc dataset; **b** Construction of multi-tissue model from GSM. Input/output (_tx) transporters were used to transport minerals, water, CO_2_, O_2_, etc., to/from external (Ext) environment. Biomass synthesis reactions (_biomass) were included to allow the model to produce biomass components which can be used to explore quantitative biomass production. Abbreviations: *Chl* chloroplast, *Mit* mitochondria, *Per* peroxisome, *Vac* vacuole, *Cyto* cytosol

Genome-scale metabolic model of *S. viridis* was constructed using the reaction dataset (version 1.0) available from Plant Metabolic Network (PMN, www.plantcyc.org [25]) as illustrated in Fig. 1. Initial reactions, genes, enzymes and pathways were obtained from PMN which provides the first draft dataset of our use. The draft dataset contains 3013 reactions and 2908 metabolites. Many of the reactions present in the dataset have missing information like, incomplete participating metabolite names, missing chemical formula, use of generic metabolite names and in few cases, wrong reaction directionality. The dataset was searched for these easily noticeable ambiguous reactions and subsequently removed. Of the 3013 reactions in the raw dataset, 961 ill-defined reactions were removed. This resulted a draft reaction dataset of our use to fed into the computational and manual reconstruction pipeline described next.

#### Sub-cellular compartmentalization

Reactions in the draft reaction dataset were compartmentalized (placing reactions in different sub-cellular organelles where they are known to occur) into five sub-cellular compartments based on the information from Arabidopsis [26] and C4 GSMs [12]. The model includes plastid, mitochondria, peroxisome and vacuole as compartments which can exchange metabolites to/from cytosol (being the ‘default’ compartment). Reactions and metabolites localized in plastid, mitochondria, peroxisome, vacuole and cytosol were distinguished by appending suffixes ‘_p’, ‘_m’, ‘_x’, ‘_v’ and ‘_c’, respectively at the end of the reaction and metabolite ids. Metabolite exchange reactions between cytosol to/from plastid, mitochondria, peroxisome and vacuole were also differentiated by appending suffixes namely, ‘_pc’, ‘_mc’, ‘_xc’ and ‘_vc’, respectively.

#### Inputs and outputs

Ten different external transport reactions (‘_tx’) were included in the model that can supply input nutrients including NO$_{3}^{-}$, ammonium (NH$_{4}^{+}$), sulfate (SO$_{4}^{2-}$), phosphate (Pi), Mg ^2+^ and photon as well as water, H ^+^, CO_2_ and O_2_ exchanges with the environment. Generic ATP consuming and NADPH oxidase steps were also included with suffix ‘_tx’ which can be used to provide maintenance requirements [26]. Biomass synthesis reactions of 40 major biomass components were included (with suffix ‘_biomass’) for amino acids, starch, sucrose, cellulose, hemi-cellulose, lignin, nucleotides, cholesterol, chlorophyll and fatty acids biosynthesis. This resulted a draft compartmentalized model for further testing.

### Curation

Curation process involved two main components - manual intervention and computational formatting. After a draft compartmentalized model was generated, we tested it for biomass production phototropically and heterotrophically under usual plant growth conditions as mentioned in “Biomass production” section. We computationally tested for any violation in thermodynamic feasibility, energetics (biomass, ATP production without energy input and transhydrogenase cycles), futile cycle and mass and charge conservation, thereafter manually corrected any wrong reaction [26–29]. In each iteration the model was improved and resulted no violation of any criteria which resulted our final model and first GSM of *S. viridis*, referred to as Sv3376 (Additional file 1) in this manuscript. Figure 1a summarizes the reconstruction process and conceptualized the formulation of Sv3376 and the multi-tissue model.

### Multi-tissue C4 metabolic model

**Fig. 2:**
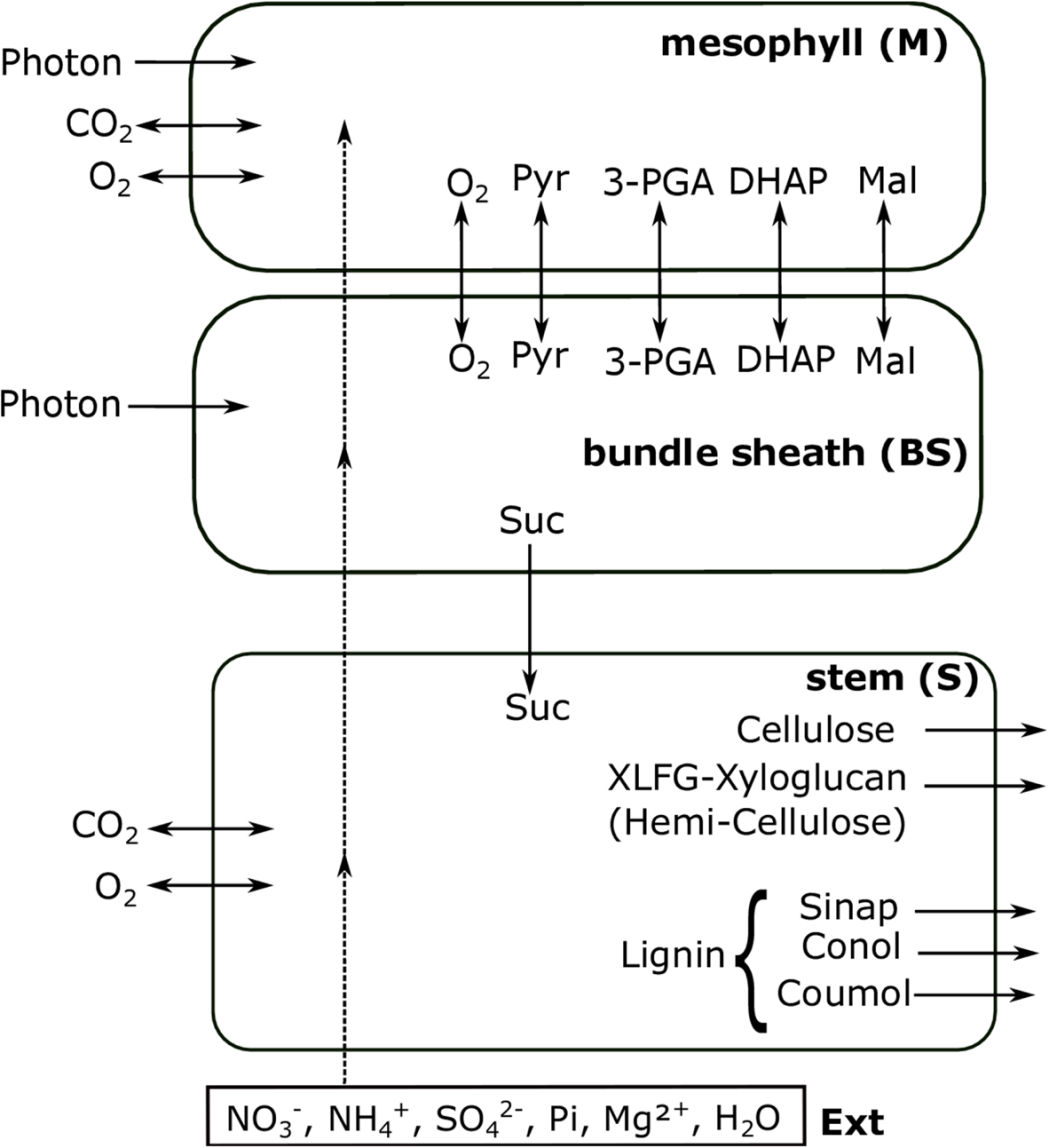
Schematic description of constraints used with the multi-tissue model to simulate the production of cellulose, hemi-cellulose (XLFG-Xyloglucan) and lignin (Sinap, sinapyl alcohol; Conol, coniferyl alcohol and Coumol, coumaryl alcohol) in stem. Gaseous exchanges of CO_2_ and O_2_ from environment were allowed from M and S cell types. All other mineral nutrients and water uptake were only allowed through S that can be distributed to BS and M (dashed linked arrow). Photon influx was allowed into both M and BS. Under two different case scenarios, model was either restricted (P _*B*_) or allowed to exchange H ^+^ from environment in the three cell types. This figure only illustrates P _*B*_ condition. Four carbon compounds, Pyr, 3-PGA, DHAP and Mal were allowed to transport between M and BS cells. O_2_ evolution to environment directly from BS was restricted but its exchange with M was allowed. Sucrose was allowed to transport from BS to S. Input of mineral nutrients from the external environment are shown (rectangle box), however only water was utilized under this simulation

Sv3376 was used to construct a multi-tissue three cell type metabolic model comprising leaf and stem tissues (Additional file 2). Leaf includes the M and BS cell types for modelling C4 photosynthesis. Each of the reactions and metabolites from Sv3376 were duplicated into three separate modules (each module being a copy of Sv3376) that represent M, BS and stem (S) cell types with suffixes ‘_MP’, ‘_BS’ and ‘_ST’, respectively (Fig. 1b). This is a widely used technique for forming multi-tissue models from a single model [30–32]. Sucrose (Suc) was allowed to be transported from leaf (i.e., from BS) to the stem. Note that we did not include the transport of amino-acids as this study focussed on the biosynthesis of lignocellulose which does not contain nitrogen. *S. viridis* uses the NADP-ME subtype of C4 carbon fixation [3], hence, exchange of malate (Mal) and pyruvate (Pyr) were allowed between M and BS. Other than this main path of carbon flow, 3-phosphoglycerate/dihydroxyacetone phosphate (3-PGA/DHAP) shuttle and oxygen exchange were also included between M and BS (Fig. 2).

### Objective used in flux balance analysis

Flux solutions were obtained by implementing flux balance analysis (FBA) [33] using CobraPy [34]. For all the simulations using Sv3376 and multi-tissue models, a bi-level optimization was used where the primary objective was set to minimize photon usage (sole photon transport for Sv3376 or total M and BS photon usages in case of multi-tissue model) and the secondary objective was to minimize total reaction flux. This bi-level optimization was implemented by first applying photon minimization as the primary objective function and solving the first optimization problem, followed by the fixing of the obtained photon flux (objective value) from the first optimization problem as constraint before solving the second optimiszation problem with the objective function of minimizing the absolute sum of all reaction fluxes. During the bi-level optimization lignocelluloses biomass values were constrained to a predetermined value in the multi-tissue model.

### Constraints used for cellulose, hemi-cellulose and lignin production in stem

Here, we obtained the flux distributions under the modelling constraints shown in Fig. 2. Multi-tissue *S. viridis* metabolic model was used to obtain the metabolic reactions and genes involved in M, BS and S cells for the production of cellulose, hemicellulosic polysaccharide XLFG xyloglucan and monolignols in stem, which are the materials used as feedstock for biofuel [35, 36]. The use of stem/straw lignocellulosic part as biofuel feedstock is of interest in C4 bioenergy plants’ research [9] and in cereal crop wastes as high lignocellulosic mass based non-food part [37].

Our model showed that sucrose from BS to S was capable of being the sole source of energy and C building block in stem for the synthesis of cellulose, hemi-cellulose and lignin. Synthesis of p-hydroxyphenyl (*H*), guaiacyl (*G*) and syringyl (*S*) units of the lignin polymer was fixed by simultaneous production of three hydroxy-cinnamyl alcohols namely, coumaryl alcohol (Coumol), coniferyl alcohol (Conol) and sinapyl alcohol (Sinap), respectively. Ratio of these monomers were fixed according to the composition of lignin found in C4 monocotyledon plants [38] to represent a similar proportion of these biomass production specific to C4 plants. We have considered the production of XLFG oligosaccharide unit of xyloglucan polymer as hemi-cellulose biosynthesis. Exchange of Mal, Pyr, DHAP and 3-PGA were allowed between M and BS. The exchange of these C compounds were set to be free, i.e. without any restriction in flux ratio or directionality. Flux through the biomass transporters were fixed according to one gram of each biomass (cellulose, hemi-cellulose and lignin) production in stem under three separate simulations. Activities of NADPH dehydrogenase and plastoquinol oxidase in chloroplast were blocked given their minor contribution in photosynthesis [39, 40]. Moreover, cytosolic NADP-ME activity was blocked in all the three cell types to effectively represent the genome encoding capability of this specific C4 sub-type plant. Also to simulate the NADP-ME subtype of C4 photosynthesis, activities of NAD-ME in mitochondria and PEPCK (phosphoenolpyruvate carboxykinase) in cytosol were blocked in all simulations. This scheme allowed CO_2_ to enter through M, form C4 intermediate (Mal) that can diffuse to BS and decarboxylate to provide CO_2_ for the Calvin-Benson (C-B) cycle. In addition to the above constraints, ribulose-1,5-bisphosphate carboxylase (RBC):oxygenase (RBO) ratio in M was set to 3:1 [31].

To investigate the effect of H ^+^ balancing on the metabolic flux predictions, flux solutions were obtained and analyzed (for both Sv3376 and multi-tissue models) with or without external H ^+^ exchanges, where the latter imposes the internal proton balance (P _*B*_) condition.

## Results

### Model properties

The genome-scale metabolic model of *S. viridis* contains 2473 reactions including external transport and inter-compartmental metabolite exchanges and 2429 metabolites. A total of 3376 unique genes are associated with the reactions and 1597 reactions have at least one gene association. All reactions in the model are balanced in atomic mass and charges, making it a robust system to predict metabolic fluxes with respect to proton balancing. A summary of the number of internal reactions and exchanges (transporters), metabolites and genes in different sections/modules of Sv3376 is given in Table 1.

**Table 1:** Summary of the number of reactions, metabolites and genes in different sections of the model

Distribution of model components			
Compartment	Internal reactions	Metabolites	Genes
Cytosol	1842	1947	2984
Mitochondria	82	125	257
Plastid	260	284	544
Peroxisome	31	55	147
Vacuole	0	18	0
Internal exchanges	Reactions	Export and biomass synthesis	Reactions
_pc	71	_tx	15
_mc	74	_biomass	40
_xc	26		
_vc	26		

Genes shown are unique to the particular compartment

We have tested the model for energy conservation to make sure that 1) no biomass can be produced without any form of energy input; 2) no ATP and NADPH generation without energy input; and 3) no transhydrogenation transferring electrons from NADH to NADPH without energy consumption. In brief, the tests were performed by constraining the model to perform one of the metabolic processes stated above while blocking all energy inputs, e.g. the generic ATPase reaction was constrained in the direction of ATP consumption with all inputs and outputs blocked to test for the presence or absence of ATP generating cycles. After several iterations of simulation and manual curation based on [26], we were able to demonstrate the absence of violation of energy conservation in the model through the inability to identify feasible solutions from the tests mentioned above. With the defined set of biomass components, our model contains 753 blocked reactions - reactions which cannot carry a non-zero flux. The number is not surprising as we have not included biomass reactions for many secondary metabolites, which is outside the scope of this work.

#### Biomass production

Sv3376 is able to produce all biomass components under phototrophic and heterotrophic conditions (Additional file 3). Under phototrophic condition, photon was the sole energy source. Heterotrophic conditions were imposed by blocking photon flux and allowing glucose (Glc) or sucrose (Suc) as the sole energy and C source. With allowed NH$_{4}^{+}$, NO$_{3}^{-}$, Pi, SO$_{4}^{2-}$, Mg ^2+^, H_2_O and H ^+^ uptake, model was able to produce all biomass components. For testing purposes, simultaneous synthesis of one mol of each biomass component were simulated under these conditions.

Relating back to realistic biomass composition, we have also tested biomass production for leaf using the biomass composition used in [14] for *S. italica* (Additional file 4). With this, we can obtain quantitative proportion of the usage of the two different nitrogen (N) sources. Model was able to utilize NH$_{4}^{+}$ and/or NO$_{3}^{-}$ as N sources. While using NH$_{4}^{+}$ or NO$_{3}^{-}$ as sole N source, the model predicted an efflux or influx of H ^+^, respectively. The model predicted a preference to use NH$_{4}^{+}$ as the sole N source for its low assimilation cost if and only if external H ^+^ exchange is possible. Given Sv3376 is proton balanced, leaf biomass synthesis was also tested under P _*B*_ condition. With the P _*B*_ constraint, both NH$_{4}^{+}$ and NO$_{3}^{-}$ were utilized at a ratio of 0.71:1 (NH$_{4}^{+}$:NO$_{3}^{-}$) to balance the charges as multiple biomass components with opposite charges need to be produced (Additional file 5). Moreover, under P _*B*_, sole NH$_{4}^{+}$ or NO$_{3}^{-}$ use generated infeasible solution (subject to fixed biomass production) indicating essential use of NH$_{4}^{+}$ :NO$_{3}^{-}$ ratio for internal ion balancing. However, if we allowed biomass components to be produced/accumulated in excess (by setting upper bound of production of biomass components as unconstrained), the use of only NH$_{4}^{+}$ or NO$_{3}^{-}$ was possible but with very high expense of energy (and excess sulfate under sole NH$_{4}^{+}$). The preferred excess biomass produced in this case were cystine or palmitate under sole NH$_{4}^{+}$ or NO$_{3}^{-}$, respectively (Additional file 5).

### Genes, reactions and pathways for the synthesis of lignocellulose biopolymers

**Fig. 3:**
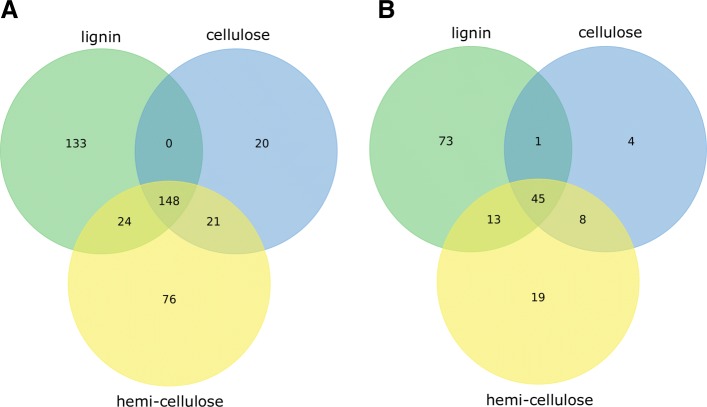
Sets of genes (**a**) and reactions (**b**) in stem involved in the production of cellulose, hemi-cellulose and lignin under P _*B*_. One gram of each biomass were fixed to produced (one at a time) and the genes/reactions expressed in stem for the biomass synthesis were used to construct the Venn diagrams

Primary cell wall of plants is a composite of biopolymers (cellulose, hemicellulose and lignin). The multi-tissue model was used to simulate the production of these biopolymers in the stem using the constraints given in Fig. 2. Figure 3 shows the number of genes (Additional file 6) and reactions (Additional file 7) in the stem which are associated with the synthesis of *S. viridis* cell wall composites. The sets of genes and reactions unique to the biosynthesis of each of the three biopolymer components were identified. Among the 73 unique reactions for lignin biosynthesis, the reactions with the highest fluxes were associated with PEP biosynthesis, and glycolytic reactions in cytosol. Highest flux among 19 reactions unique to XLFG xylogucan production include the biosynthesis of UDP-D-xylose (Additional file 8). We identified 45 reactions common for the synthesis of all three biopolymer components, which mainly include sucrose degradation for energy generation and providing C skeleton for synthesis of the biopolymers. With regards to leaf metabolism, there was not much variation occurred in the selection of genes/reactions (data not shown) in M and BS as for their common metabolic activity for carbon assimilation and sucrose synthesis.

**Fig. 4:**
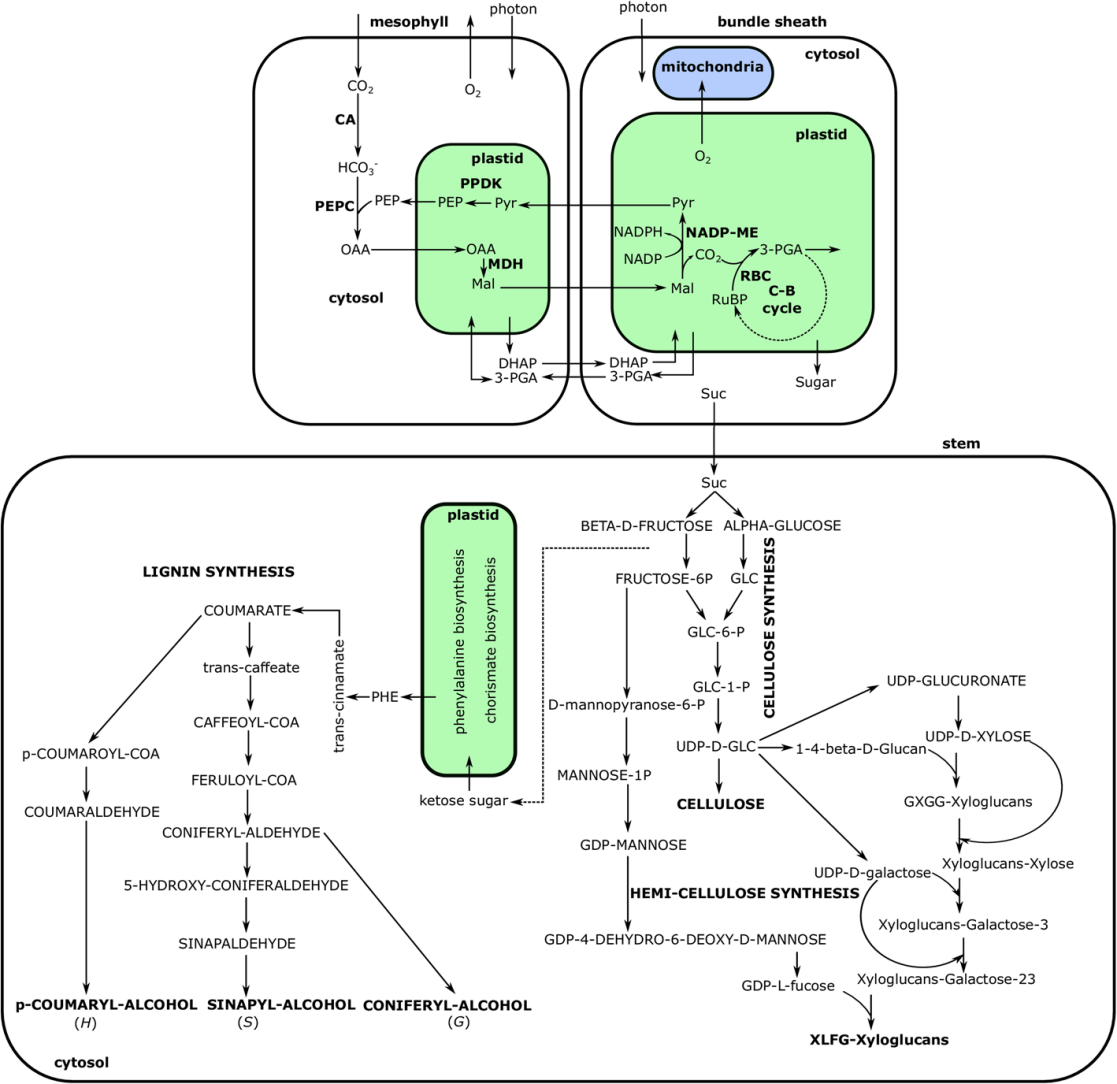
Main biosynthetic pathway for the biosynthesis of cellulose, XLFG-xylogucan and monolignols i.e., coumaryl, coniferyl and sinapyl alcohols under P _*B*_. The flux map shown here for M and BS is common to all the three biomass synthesis scenarios. Main routes for lignin, cellulose and XLFG-xylogucan biosynthesis are shown in the stem. Abbreviations: CA, carbonic anhydrase; PEPC, phosphoenolpyruvate carboxylase; MDH, malate dehydrogenase, PPDK, pyruvateorthophosphate-dikinase, RBC, ribulose-bisphosphate-carboxylase

Table 2 shows that the photon demand for the biosynthesis of one gram lignin is higher than that of cellulose and hemi-cellulose. Almost double the amount of sucrose was required from the leaf to the stem for lignin biosynthesis compared to the biosynthesis of other two polysaccharides. Photon requirements and the amount of sucrose transported from leaf to stem were similar for the synthesis of one gram cellulose and hemi-cellulose. The major metabolic fluxes predicted by the multi-tissue model for biopolymer synthesis include the utilization of NADP-ME subtype of C4 photosyntheis in the leaf, sucrose synthesis in BS cell, sucrose transport to the stem and utilization for cell wall components’ synthesis (Fig. 4). The monolignol biosynthetic pathway from phenylalanine (PHE) was predicted by the model, which was found to be favored in angiosperms by previous experimental studies [41] and modelling work in *S. italica* [14]. Similarly, the model was able to predict the involvement of glucan and different glycosyl transferases in the biosynthesis of XLFG xyloglucan subunit, as demonstrated in previous studies [42, 43].

**Table 2:** Flux values for the production of one gram of each cellulose, hemi-cellulose and lignin using constraints shown in Fig. 2

Flux (mol g ^−1^ DW)	cellulose		hemi-cellulose		lignin	
Sucrose from leaf to stem	3.29 ×10^−3^		3.34 ×10^−3^		7.02 ×10^−3^	
	M	BS	M	BS	M	BS
Individual photon demand	0.381	0.104	0.386	0.106	0.811	0.222
Total photon demand	0.485		0.492		1.033	

M and BS denote values for mesophyll and bundle sheath respectively. Values are rounded off to three decimal places. All values presented are in mol g ^−1^ dry weight (DW) of respective biomass produced in an arbitrary time

#### Proton balancing is important for predicting metabolite exchanges between M and BS cells

The flux distribution in M and BS under the constraints of producing lignocellulosic biomass (Fig. 2) utilized the 3-PGA/DHAP shuttle as a secondary exchange of carbon in addition to the Pyr/Mal shuttle in the C4 pathway. However, this exchange was deactivated when the P _*B*_ constraint was relaxed, i.e. with allowed exchanges of H ^+^ from M and BS to outer environment (data not shown). Moreover, the scenario of relaxed P _*B*_ constraint required more photon through BS than M unlike the high photon demand in M compared to BS for the simulations under P _*B*_ (Table 2). Interestingly, oxygen evolved from the chloroplast in BS cell was all used up in the mitochondria, with no oxygen transport between M and BS cells under P _*B*_ constraint. By relaxing the P _*B*_ constraint, the model predicted an oxygen flux from BS to M, which could be an effect of the change in 3-PGA/DHAP shuttle in transporting reducing power. Our results showed that the constraint of balancing internal H ^+^ production and utilization (P _*B*_) influenced the model predictions on the metabolite shuttles between M and BS cells, demonstrating a previously unnoticed role of the 3-PGA/DHAP shuttle in balancing the utilization and production of protons, and possibly oxygen flux, between M and BS cells.

## Discussion

### Comparison with other plant models

Recently, a genome-scale metabolic model of *S. italica* was developed using a C4 plant specific metabolic model framework, C4GEM, [12] as an initial genome-scale reconstruction [14]. The *S. italica* model was used to study different metabolic characteristics in young and mature stem/leaf phytomers by integrating multi-omics data. The *S. italica* reconstruction was mainly used to develop annotation and multi-omics protocols for systems analysis which is also applicable to our *S. viridis* metabolic model to observe many C4 metabolic traits using ‘ideal lab organism’, similar to how *A. thaliana* has been used to study C3 plants [4]. Apart from the different varieties, the focus of [14] was on integration of omics data, whereas the aims of our study were to test the importance of the P _*B*_ constraint, lignocellulose gene mapping and biomass production. Prior to this study, a mass and charge balanced maize (*Zea mays*) genome-scale metabolic model [44] was derived from maize genome, associations with AraGEM [45] and other C3 and C4 species. The model was applied to investigate biomass production under different physiological states and mutant conditions. Conceptually, our work is closer to this study for lignin biosynthesis observation but differs with regards to species and specific findings such as the role of ammonium-nitrate mixture to effectively balance cation-anion for energy efficient growth, internal charge balancing on 3-PGA/DHAP shuttle and favorable metabolic activities of mesophyll and bundle sheath cells. Another maize genome-scale model was developed based on maize genome resource data (CornCyc, PMN), to address non-linear relationship and response of C4 systems to perturbations [24]. However, to date no *S. viridis* genome-scale metabolic reconstruction is available that merits potential for direct comparison.

### Identification of target genes can accelerate future bioengineering research

Discovery of genes controlling the process of C4 photosynthesis in *S. viridis* accompanying with modern computational and experimental techniques has the potential to improve C4 engineering that is directly related to improving crop and biofuel production [8]. At the time when global food production needs to be improved for the rising demand, the engineering of C4 photosynthesis is likely to a promising solution. A better understanding of the underlying genetic mechanisms will certainly help in accelerating the engineering of C4 differentiations from anatomy to viable pathways [10]. To speed up this process, a metabolic model of *S. viridis* can be a useful tool for identifying target genes for achieving several desired metabolic actions as demonstrated by our model’s ability to predict genes involved in the biosynthesis of individual biomass components (Additional file 6). Moreover, with the future application of *S. viridis* in synthetic biological research in mind, our model can become an economical tool to identify quantitative metabolic variation under multiple gene target’s partial inhibition rather than complete inhibition of a single target which at least proved efficient for drug design with minimal side effects [46]. Bioethanol industry rely on cost effective source of biomass and their continuous supply [9], thus understanding the metabolism of C4 plants, which are efficient in terms of N and water usage, through computational and experimental research can provide insights into engineering better feedstock crops. With the use of large-scale metabolic modeling, systems-level effects can be observed in the perspective of entire metabolic network, i.e. a holistic view of reactions, inter-compartmental exchanges to biomass synthesis across space and time with multi-tissue dynamic modeling [30]. At the same time, *S. viridis* also being a model C4 organism can be used for *in-vivo* validation for molecular breeding outcomes and target gene identification to improved genotypes [4].

### Efficient metabolic activity favors utilization of NH$_{4}^{+}$-NO$_{3}^{-}$ ratios

The mass and charge balanced Sv3376 favors the use of both NH$_{4}^{+}$ and NO$_{3}^{-}$ under P _*B*_ condition while simulating to produce biomass components as given for *S. italica* (Additional file 5). While there is an energetic cost for reducing NO$_{3}^{-}$ to NH$_{4}^{+}$, our model showed that an uptake of NO$_{3}^{-}$ could be important as blocking NO$_{3}^{-}$ uptake under P _*B*_ required an 117% increase in N (NH$_{4}^{+}$) use and corresponding 65.2% increase in photon usage as model balanced the cation by taking up excess SO$_{4}^{2-}$ (under unrestricted upper bound in leaf biomass proportions and lower bound fixed to experimental values for all biomass components; Additional file 5). The surplus N and S lead to increased cysteine (Cys) accumulation (6.85 mmol, previously it was 0.045 mmol). In reality the excess proton from pure use of NH$_{4}^{+}$ could be potentially be transported to the roots and be excreted, for example high external NH$_{4}^{+}$ concentration forces the N uptake in cationic form, leading to secretion of H ^+^ from root to balance the process [19]. This behavior of H ^+^ secretion to balance the internal cation-anion when NH$_{4}^{+}$ used as the sole N source was predicted by our model when the P _*B*_ constraint was relaxed. Meanwhile, with the P _*B*_ constraint, our model demonstrated a potential role of using NH$_{4}^{+}$ and NO$_{3}^{-}$ for balancing cation-anion in a relatively closed system despite the costs of NO$_{3}^{-}$ uptake and assimilation are higher. Experimental studies also suggest that plants could maintain desired pH range and cation-to-anion uptake ratio by adjusting NH$_{4}^{+}$:NO$_{3}^{-}$ from total N supplied [47]. The use of 1:3 ratio for NH$_{4}^{+}$:NO$_{3}^{-}$ was shown to be beneficial for plant growth, yield, and quality of fruits [47] and presumably it also save the overall energy budget. Therefore, a plant’s physiological and environmental conditions may favor different strategies over assumed ideal cases and for this we need mass and charge balanced models to accurate predict such metabolic adaptations. From a metabolic engineering perspective, one needs to consider the balancing of charges as our model demonstrated that some biomass components, e.g. cysteine and me-thionine, cannot be produced individually without producing other metabolites under P _*B*_, but not with relaxed P _*B*_, due to the requirement for balancing charges (data not shown).

### Role of internal proton balancing in regulating the use of enzymes

In NADP-ME subtype of C4 species, [48] observed a limited PSII activity in the BS chloroplasts, leading to lower oxygen production in BS cells. This is consistent with our simulations under the P _*B*_ constraint where PSII activity was lower in BS than in M (Additional file 8). Interestingly, oxygen diffusion from BS to M was inactive (Fig. 4) under the P _*B*_ constraint, while oxygen transport was activated when we allowed H ^+^ exchanges in M and BS. This suggests that the requirement of H ^+^ balancing (P _*B*_) could impose a stoichiometric constraint in reducing PSII activity in BS. It would be interesting to test if the same applies to other C4 subtypes.

Lower PSII activity can limit the production of reduction equivalents in BS and necessitates the shuttling of 3-PGA/DHAP to supply NADPH for CO_2_ assimilation. Thus, in the condition of balancing in internal H ^+^ production and consumption, we observed a division of labor where M was mainly utilized for energy generation and BS was used for CO_2_ (re)fixation with reduced activity of some enzymes for better utilization of enzymatic machinery. For instance, flux distribution in BS plastid shows a significant reduction in the flux from H ^+^ consuming reactions (ferredoxin NADP reductase, plastoquinol-plastocyanin reductase and reversible GAP-dehydrogenase), whereas plastidial ATP synthase activity was also reduced generating reduced amount of ATP and H ^+^ in BS. Thus, to satisfy the energy demand, photon influx in M was higher than BS, which may coincide with the evolution of Kranz anatomy in C4 leaves.

### 3-PGA/DHAP shuttle - an essential prerequisite for the specific roles of mesophyll and bundle sheath cells

Our model highlighted the requirement of 3-PGA/triosephosphate shuttle between M and BS under proton balance condition. This 3-carbon sugar phosphate shuttle is known to have a role in the net transfer of reducing power between M and BS and might be able to control energetic load on mesophyll chloroplast [49]. Metabolic modeling provides an opportunity to test its distinct role and the consequences of its absence.

For this, an additional simulation was conducted by modifying the H ^+^ transport mechanism in the model. In this scenario, H ^+^ exchanges directly from Ext to M and BS were blocked (while H ^+^ transport from Ext ⇔S was allowed) and two new transporters were temporally added from stem to bundle sheath and from bundle sheath to mesophyll (S ⇔*BS*⇔M). With these modifications, the model preferred to use H ^+^ transport in the direction from M to BS in place of the 3-PGA/DHAP shuttle (Additional file 9). Moreover, H ^+^ uptake and transport to BS (Ext ⇔*S*⇔BS) was also inactive and similar photon was utilized as original model (sum of total photon demands given in Table 2). However, a closer look at the individual photon demands in M (0.892 mol photons) and BS (1.117 mol photons) shows that it requires more photon to be used in BS when the model utilizes H ^+^ exchange between M and BS in this scenario, while this was opposite in case of 3-PGA/DHAP shuttle (see Table 2). Our modeling results suggest that there is a link between the photon demands in BS and M, PSII activities in BS and M, the use of 3-PGA/triosephosphate shuttle between BS and M and the balancing and transport of H ^+^ between BS and M.

Continuing with the scenario of modified proton transport, when 3-PGA/DHAP shuttle and H ^+^ exchange between M and BS were simultaneously blocked, the model predicted an 82% increase in photon demand and an unconventional reverse exchange of pyruvate and malate between M and BS (pyruvate transported from M to BS and malate transported from BS to M; Additional file 9). Moreover, NADP-ME was deactivated in BS and main carbon fixation was occurred in M with active photorespiration as in C3 plants. This demonstrates the importance of balancing protons between M and BS. From our modelling results, we have uncovered a previously unrecognized role of the 3-PGA/DHAP shuttle in the H ^+^ balancing between M and BS.

## Conclusion

The mass and charge balanced genome-scale metabolic model of ‘model C4 species’, *S. viridis*, presented in this study has the potential to accelerate hypothesis generation and testing. The model identified some metabolic features not previously reported, such as the role of the 3-PGA/DHAP shuttle in balancing protons between M and BS cells. A metabolic model of *S. viridis* with extensive reaction to gene mapping presented in this study could be a useful tool for the identification of target genes for metabolic engineering, and can be applied to approaches with “omics” data integration. These approaches have applications in crop and feedstock improvement and transferring C4 metabolic traits to C3 plants for better N and water usage. Apart, from biotechnological research, understanding of the fundamental plant behavior for nutrient usage and cell-type specific metabolic roles require a mass and charge balanced model as demonstrated in this study.

## Additional files

Additional file 1 Genome-scale metabolic model of *Setaria viridis* (Sv3376). Reactions, metabolites, chemical formulae, pathways, EC numbers and gene association of reactions. (XLS 1727 kb)

Additional file 2 Multi-tissue metabolic model of *S. viridis* comprising leaf and stem tissues. Multi-tissue version of Sv3376 to simulate C4 photosynthesis. (XLS 3944 kb)

Additional file 3 Biomass production using Sv3376 under phototrophic and heterotrophic condition. One mol of each biomass production in phototrophic and heterotrophic condition under allowed and blocked proton. (XLS 131 kb)

Additional file 4 Leaf biomass composition. Biomass proportions used to simulate the production of one gram of *Setaria viridis* leaf biomass. (XLSX 14 kb)

Additional file 5 Biomass production using Sv3376 for different N sources. Flux distributions for the production of one gram leaf biomass i) with P _*B*_ and both nitrate and ammonium, ii) with P _*B*_ constrained and ammonium as sole N source and iii) with P _*B*_ constrained and nitrate as sole N source. (XLSX 14, 565 kb)

Additional file 6 Genes expressed in stem. Different set of genes expressed in stem for the production of cellulose, hemi-cellulose and lignin. These sets represent the genes which are common and/or unique to different biomass production. Phytozome (phytozome.jgi.doe.gov/pz/portal.html) database identifiers are included for these genes. (XLS 135 kb)

Additional file 7 Reactions activated in stem. Different set of reactions in stem for the production of cellulose, hemi-cellulose and lignin. These sets represent the reactions associated with the genes (Additional file 6) which are common and/or unique to different biomass production. (XLS 43 kb)

Additional file 8 Flux distributions for the production of cellulose, hemi-cellulose and lignin. Flux solutions obtained using the multi-tissue model for the production of cellulose, hemi-cellulose and lignin in stem. (XLSX 24 kb)

Additional file 9 Flux distributions under modified proton transport mechanism between M, BS and S. Flux solutions obtained when proton transport mechanism temporarily modified in the multi-tissue model. Two flux solutions provided: proton and 3-PGA/DHAP shuttle allowed between M and BS and when both exchanges were blocked. (XLS 68 kb)

## Data Availability

All data generated or analysed during this study are included in this published article and its supplementary information files. See online version of the article and additional files on BMC website.
